# Fast Fabrication of Solid-State Nanopores for DNA Molecule Analysis

**DOI:** 10.3390/nano11092450

**Published:** 2021-09-20

**Authors:** Yin Zhang, Dexian Ma, Zengdao Gu, Lijian Zhan, Jingjie Sha

**Affiliations:** 1 Jiangsu Key Laboratory for Design and Manufacture of Micro-Nano Biomedical Instruments, School of Mechanical Engineering, Southeast University, Nanjing 211189, China; a1048694537@163.com (D.M.); 220190300@seu.edu.cn (Z.G.); lijianzhan@seu.edu.cn (L.Z.); 2 China Aerospace Science & Industry Nanjing Chenguang Group, Nanjing 210006, China

**Keywords:** solid-state nanopore, dielectric breakdown, biosensor, DNA detection

## Abstract

Solid-state nanopores have been developed as a prominent tool for single molecule analysis in versatile applications. Although controlled dielectric breakdown (CDB) is the most accessible method for a single nanopore fabrication, it is still necessary to improve the fabrication efficiency and avoid the generation of multiple nanopores. In this work, we treated the SiNx membranes in the air–plasma before the CDB process, which shortened the time-to-pore-formation by orders of magnitude. λ-DNA translocation experiments validated the functionality of the pore and substantiated the presence of only a single pore on the membrane. Our fabricated pore could also be successfully used to detect short single-stranded DNA (ssDNA) fragments. Using to ionic current signals, ssDNA fragments with different lengths could be clearly distinguished. These results will provide a valuable reference for the nanopore fabrication and DNA analysis.

## 1. Introduction

Over the past three decades, nanopores have been developed as a class of powerful single-molecule sensors and wildly utilized in biophysics, chemistry, biology, and medicine [1,2,3,4,5,6,7]. Nanopore detection is based on the measurement of ionic current modulation from individual analytes’ translocation through the nanopore, which reveals the physical characteristics of the analytes. To facilitate nanopore detection, the size of the nanopores must be similar to that of the analytes. Because of the atomic precision of the required dimensions, engineered protein nanopores achieved the first read of DNA sequences [8] and subsequently blossomed into a commercial sequencing platform [9]. However, protein nanopores also have some inherent drawbacks, such as the instability of the supported lipid bilayer membrane and the fixed pore size/geometry. Solid-state nanopores have emerged as a versatile substitute for protein nanopores because of the advantages of robustness, durability, and tunable size [10,11]. Moreover, because of the integration compatibility of solid-state nanopores with various platforms [12], resistive-pulse sensing is easily coupled with other detection modalities based on plasmonic sensing [13], fluorescence spectroscopy [14], force spectroscopy [15], field effect transistors [16,17,18] and quantum tunnelling [19]. To date, solid-state nanopores have been used broadly for the detection of RNA [20,21], proteins [22,23], viruses [24], exosomes [25], and some other bionanoparticles [26,27], as well as DNA [28]. Solid-state nanopores also enable measuring the physical characteristics of nanomaterials, including not only the size but also the shape [29] and stiffness [30]. At the outset, approaches for fabricating solid-state pores with diameters below 10 nm mainly relied on focused ion [31] or electron [32] beams. However, these techniques require high-cost instrumentation and advanced skill, which limits the availability of nanopore analytics to a wider range of applications [33].

To overcome this bottleneck, Kwok et al. [34,35] pioneered an alternate simple fabrication technique via controlled dielectric breakdown (CDB) of an insulating silicon nitride membrane in aqueous solution. Subsequently, some research groups sought to improve CDB by using different breakdown voltage profiles to automatically control the pore size [36] and pore shape [37]. Now, CDB has become a popular approach to creating nanopores with angstrom-level precision in a variety of materials such as SiN_x_ [38], SiO_2_ [39], and HfO_2_ [40]. In a typical CDB process, the external electric field applied across an insulating membrane is in a range of 0.4–1 V/nm. It has been reported that high electric fields increase the risk of generating multiple pores [41,42,43], whereas low transmembrane potential (<0.5 V/nm) leads to the exponential extension of time-to-pore formation on the order of several thousand seconds [34,44] and results in low throughput of pore fabrication. Membrane properties also play an active role in determining the time to breakdown. Carlsen et al. [41] employed a helium ion microscope to selectively thin a SiN membrane and induce defects, which reduced the time of CDB. Mayer’s group [42] proposed a CDB platform assisted by a focused laser beam, which was able to locally accelerate defect formation and improve pore formation efficiency. However, these approaches still required extra specialized facilities and trained operators. The pH of the solution is another major factor that affects time-to-pore-formation. Tabard-Cossa’s group [34] demonstrated that proton incorporation or hole injection leads to an impact-ionization-induced avalanche. Therefore, low pH speeds up the dielectric breakdown process. However, most nanopore detection experiments need to be conducted in neutral solution. Furthermore, the character of the solution–membrane interface is also crucial in the CDB process. Air/oxygen plasma is often used to clean and hydrophilize SiN membranes. Although some researchers treated the membrane by plasma before CDB [34,36,41], they did not realize the importance of this treatment in improving CDB efficiency. In this paper, we sought to explore the effects of plasma treatment on time-to-pore-formation and breakdown voltage. Here, the functionality of CDB-fabricated nanopores was validated by λ-DNA translocation experiments. In addition, we detected short ssDNAs and investigated their electrokinetic behaviors using pores of various sizes.

## 2. Materials and Methods

**Figure 1:**
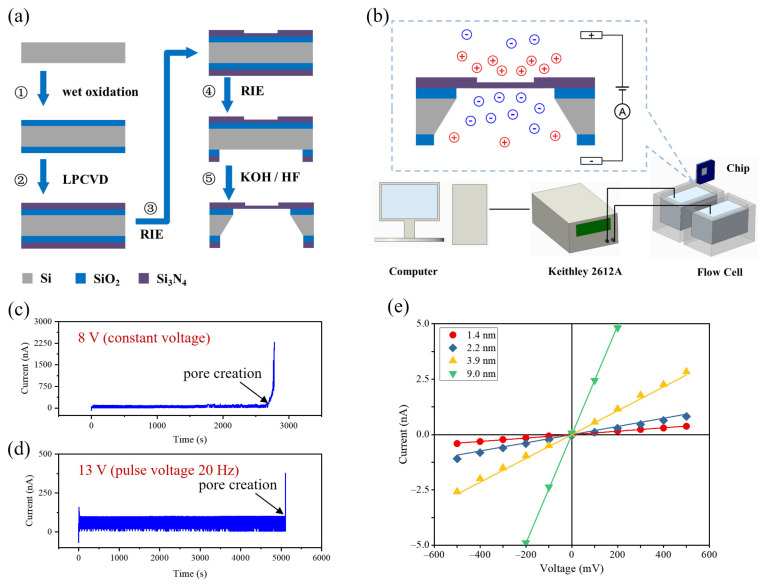
Schematic illustrations of (**a**) the chip fabrication process; (**b**) the nanopore fabrication setup; leakage current traces at (**c**) 8 V constant voltage and (**d**) 13 V pulse voltage on a 20 nm-thick SiN_x_ membrane in 1 M KCl at pH 8. (**e**) I–V curves for 4 nanopores with various diameters.

Chip fabrication: as shown in Figure 1a, the nanopore chip fabrication processes began with the growth of 1 μm-thick SiO_2_ insulating layers on both sides of a Si wafer (RDMICRO Inc., Suzhou, China) by wet oxidation. The next step was the deposition of 60 nm-thick low-stress SiN_x_ membranes on the SiO_2_ layers by low-pressure chemical vapor deposition (LPCVD). Then, a 2 μm-diameter circular region of the top SiN_x_ membrane was thinned from 60 to 20 nm by UV lithography (UVL) and reactive ion etching (RIE). After that, a 323 μm × 323 μm square etch window was opened on the bottom deposition layer by UVL and RIE. Finally, the wafer went through a KOH wet etching process and a HF wet etching process to remove the Si substrate and SiO_2_ layer, respectively.

Fabrication of nanopores by CDB: a nanopore chip was treated in an air–plasma cleaner with a radiofrequency power of 30 W for 30 s on both sides and then mounted into a custom-built polymethylmethacrylate (PMMA) flow cell. The *cis* chamber and *trans* chamber of the flow cell were filled with degassed and filtered 1 M KCl solution (buffered with 10 mM Tris-HCl and 1 mM EDTA to pH 8) and separated by the chip. As shown in Figure 1b, two Ag/AgCl electrodes were connected to a sourcemeter (Keithley 2612A, Tektronix Inc., Beaverton, OR, USA) and immersed into two electrolyte compartments to set up a constant or pulse voltage and monitor the induced leakage current with a sampling rate of 20 Hz. The CDB procedure was controlled by a custom-designed program. To maintain accurate control over the nanopore size, we employed the applied square pulse voltage at 20 Hz to minimize the amount of membrane material removed during each leakage current feedback loop.

Detection of DNA translocations through nanopores: DNA samples were purchased from Sangon Biotech Co., Ltd. (Shanghai, China) and added in the *cis* chamber. Ionic current traces were recorded by a resistive feedback amplifier (Axon MultiClamp 700B, Molecular Devices LLC, San Jose, CA, USA) at 250 kHz with a 10 kHz low-pass filter. In the DNA detection experiments, the *trans* chamber was electrically grounded, and positive or negative potentials were applied to the *cis* chamber. All nanopore-based detection experiments were conducted inside a dark Faraday cage.

## 3. Results and Discussion

During the CDB process, the transmembrane potential induced defect accumulation in the SiN_x_ membrane. Once a subnanometer diameter pore was created, the leakage current suddenly increased, as shown in Figure 1c,d. After observation of this initial pore creation event, the electric field was turned off immediately or reduced to slowly enlarge the nanopore. Figure 1e shows the measured current–voltage (I–V) curves of various-sized nanopores fabricated by CDB in 1 M KCl solution. The effective diameter of a cylindrical nanopore can be calculated from the ionic conductance of the pore, *G*, based on Equation (1) [45]:

(1) $$G=\sigma {\left(\frac{4l}{\pi d_{pore}^{2}}+\frac{1}{d_{pore}}\right)}^{-1}$$

where *σ* is the bulk electrolytic conductivity (for 1 M KCl, *σ* = 10.5 S/m at 23 °C) and *l* is the length of the nanopore.

**Figure 2:**
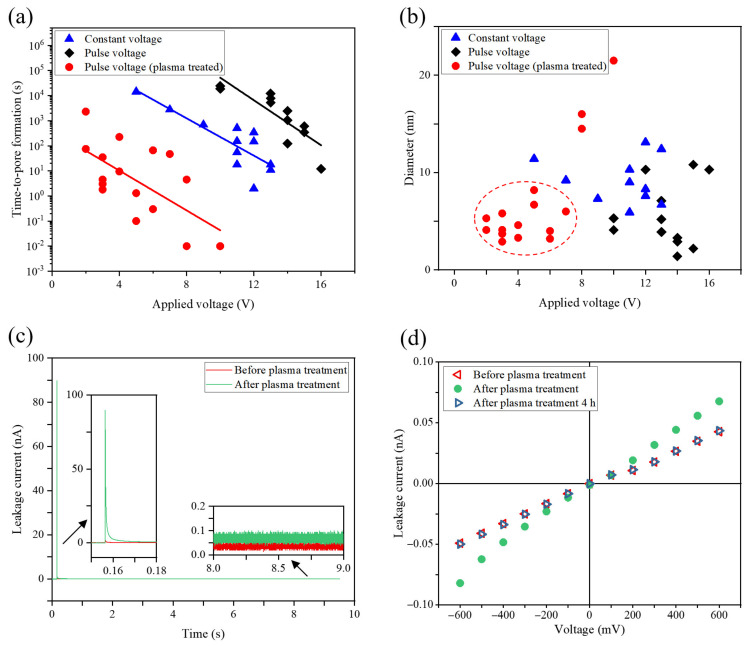
(**a**) Semi-log plot of time-to-pore-fabrication as a function of applied voltage for nanopores fabricated in 20 nm-thick SiN_x_ membranes in 1 M KCl solution; (**b**) scatter plot of pore diameter versus applied voltage; (**c**) leakage current trace of a SiN_x_ membrane before and after plasma treatment at a 600 mV applied voltage; (**d**) I–V curves for the SiN_x_ membrane.

Here, we fabricated nanopores by using constant and pulse voltage profiles. A set of chips was treated in plasma for 30 s at 30 W before the CDB procedure. As shown in Figure 2a, the time-to-pore-formation exponentially decreased as the applied voltage increased regardless of other conditions, which is consistent with previously reported work [34,44]. Interestingly, the time-to-pore-formation under constant voltage was more than an order of magnitude longer than that under pulse voltage, although the duty cycle of the pulse voltage was 50%. Furthermore, after plasma treatment, the breakdown process sped up by orders of magnitude and required lower breakdown voltage. As shown in Figure 2b, the use of voltages in a range 2–7 V could steadily produce nanopores with sub-10 nm diameters. When the transmembrane voltage reached 8 V or above, instantaneous breakdown events were observed, and the resulting pore size was larger than 15 nm. Previous studies have represented the mechanism of CDB as follows: (i) oxidation reactions of chloride ions (or reduction reactions of hydrogen ions) occur at the solution–membrane interface to supply (or remove) electrons; (ii) the electrons travel through charge traps in the SiN_x_ membrane, which forms a highly conductive path and increases damage due to Joule heating [43,46,47]. Here, we measured the leakage current traces for the SiN_x_ membrane before and after plasma treatment at a 600 mV applied voltage. Figure 2c shows the leakage current increased after plasma treatment. We supposed that the influence of plasma treatment on the current flow derived from two aspects. On one hand, air plasma markedly hydrophilized the surface of SiN_x_ membrane, which may enhance oxidation/reduction reactions at solution–membrane interface. On the other hand, plasma exposure also induced damage in the membrane [48,49], which may facilitate electron transport across the membrane. To confirm these hypotheses, we exposed the plasma-treated SiN_x_ membrane to ambient atmosphere for 4 h and measured the I–V curve again. As shown in Figure 2d, the conductance of the SiN_x_ membrane recovered to its pretreatment level after storage in air for 4 h, as did the nature of the membrane–liquid interface. This indicates that the dominant factor by which plasma treatment sped up the CDB process was enhancing oxidation/reduction reactions rather than inducing defects in the membrane.

**Figure 3:**
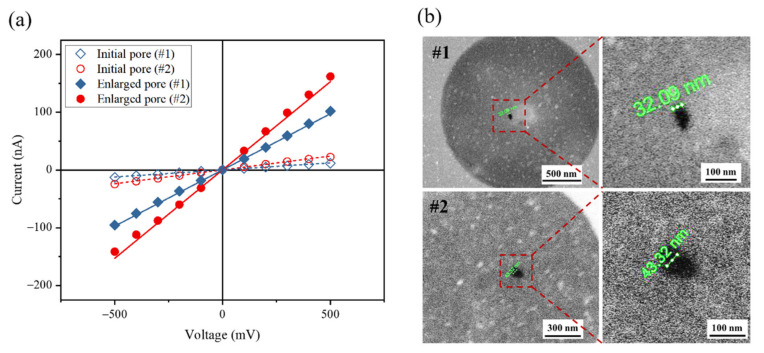
(**a**) I–V curves of nanopores before and after enlargement breakdown; (**b**) SEM images of nanopores after enlargement breakdown.

In order to obtain larger-sized pores with controllable diameters, bipolar square pulses were employed after pore creation to enlarge the size of plasma-treated pores. By applying a 3 V transmembrane potential, pore diameter could enlarge to >30 nm within 10 min. Figure 3a shows the I–V curves of two nanopores before and after enlargement breakdown. Based on Equation (1), it was estimated that: the initial size of pore #1 was 9 nm, that of pore #2 was 13.3 nm, the final size of pore #1 was 32.9 nm, and that of pore #2 was 45.5 nm. Previous studies have demonstrated the formation of multiple pores during the enlargement process [50,51,52]. In our study, there was a thinned region in the SiN_x_ membrane where the electric field was stronger and additional pores may have been created [41]. However, scanning electron microscope (SEM) images (Figure 3b) indicate that there was only a single nanopore in the thinned area after enlargement breakdown, even though the 3 V transmembrane potential was able to break down the plasma-treated membrane. Furthermore, the measured pore diameters were comparable to the calculated value, which further confirmed that only a single pore existed on the SiN_x_ membrane. There are two reasons why no other pores were formed. First, after a nanopore formed, the electric field concentrated in the pore, which caused the electric field strength to reduce in other regions of the membrane. Second, in our work, the expansion electric field strength was 0.15 V/nm, which is less than the strength of the fields used in previous studies that resulted in multiple pore formation [41,50,52].

**Figure 4:**
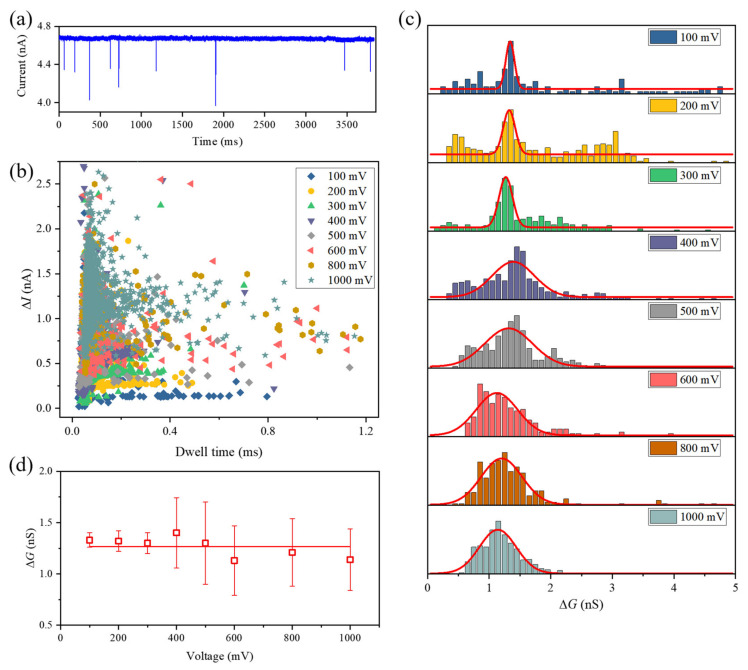
(**a**) Ionic current trace of the translocation of λ-DNA molecules through an 8 nm-diameter nanopore in 1 M KCl with an applied voltage of 200 mV; (**b**) scatter plot of dwell time versus current blockade at various voltages, ranging from 100 to 1000 mV; (**c**) histograms of ∆*G* for λ-DNA molecule translocation through the nanopore. Solid red lines represent the fit of a Gaussian distribution to the experimental data, from which we extracted the mean ∆*G*; (**d**) mean ∆*G* of translocation events with various voltages.

To validate the functionality of our CDB-fabricated nanopores, we first detected λ-DNA molecules by using an 8 nm-diameter nanopore in 1 M KCl. The ionic current trace at 200 mV bias voltage is shown in Figure 4a, in which pulse signals indicate the events corresponding to λ-DNA molecule translocation through the pore from the *cis* chamber to the *trans* chamber. Figure 4b presents the scatter plot of translocation dwell time versus current blockade, ∆*I*, with various voltages ranging from 100 to 1000 mV. As we expect, ∆*I* generally increased with the applied voltage. Histograms of conductance change ∆*G* caused by λ-DNA molecule translocation through the pore are plotted in Figure 4c. Red curves are Gaussian fits to the ∆*G* distributions. As shown in Figure 4d, the fitting peaks of ∆*G* almost remained constant, with a mean value of 1.27 nS. These results exhibit the stability of our CDB-fabricated nanopore at every applied voltage. ∆*G* also can be estimated by Kowalczyk’s model [45]:

(2) $$\Delta G=G\left(d_{pore}\right)-G\left(d_{with\;DNA}\right)$$

where $d_{with\;DNA}=\sqrt{d_{pore}^{2}-d_{DNA}^{2}}$ is the effective diameter of the nanopore when a DNA molecule is in the pore. Taking *d_DNA_* = 2.2 nm and *d_pore_* = 8 nm, the calculated value of ∆*G* is 1.35 nS, which corresponds closely to the measured ∆*G*. This further verifies the presence of a single pore on the membrane.

We next conducted translocation experiments of 20 nt and 50 nt short single-stranded DNA (ssDNA) fragments by using a 6 nm-diameter pore and an 11.5 nm-diameter pore in 1 M KCl. The ssDNA fragments were added into the *cis* chamber, where +400 or −400 mV voltage was applied. The *trans* chamber was electrically grounded. Notably, the ±400 mV applied voltage slowly enlarged the pore size during the ssDNA detection experiments. However, as estimated by Equation (1), the pore diameter grew only by several angstroms after more than 30 min of nanopore sensing. This barely affected the blockade current signals caused by DNA translocation events.

**Figure 5:**
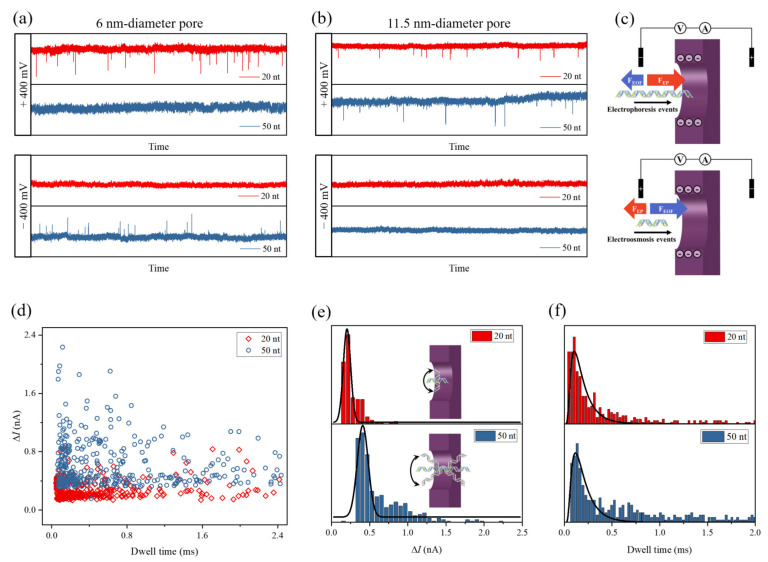
Ionic current traces with the translocations of 20 nt (red) and 50 nt (blue) ssDNA fragments through (**a**) 6 nm and (**b**) 11.5 nm diameter nanopores. The ssDNA fragments were added in the *cis* chamber filled with 1 M KCl solution. The applied voltage on the *cis* chamber was +400 or −400 mV, and the *trans* chamber was electrically grounded; (**c**) schematic of an ssDNA transport through the pore, governed by the competition of *F_EO_* and *F_EP_*; (**d**) scatter plot of dwell time versus current blockade for 20 nt and 50 nt ssDNAs electroosmotically transported through a 11.5 nm-diameter pore; (**e**) histograms of ∆*I* for electroosmotic translocation events, which were fitted by Gaussian distribution; (**f**) histograms of dwell time for electroosmotic translocation events, which were fitted by first-passage-time distribution.

As shown in Figure 5a, the translocation events of 20 nt ssDNAs were observed in the ionic current trace when the applied voltage was +400 mV. It is well known that the surface of ssDNA and SiN_x_ is negatively charged in neutral solution. The direction of electrophoretic force (*F_EP_*) acting on the ssDNAs was from the *trans* side to the *cis* side. The electroosmotic flow (EOF), as well as the 20 nt ssDNAs, moved in the same direction as the electric field. Therefore, the translocation of 20 nt ssDNAs through the 6 nm nanopore was dominated by electroosmotic force (*F_EO_*). Oppositely, the 50 nt ssDNA molecules could be driven through the same pore only by *F_EP_* at −400 mV. Therefore, the electrokinetic behaviors of ssDNA through a nanopore are governed by the competition of *F_EO_* and *F_EP_*, as shown in Figure 5c. When this is the case, ssDNAs with different length can be easily distinguished and separated by nanopore. However, for the 11.5 nm-diameter pore, both 20 nt and 50 nt ssDNAs were driven through the pore by EOF, as shown in Figure 5b. The relationship among ssDNA direction of movement, ssDNA length, and pore size has been well studied in our previous work [53]. In order to further analyze the electroosmotic events, we plotted a scatter chart of dwell time versus current blockade for 20 nt and 50 nt ssDNAs transporting through the 11.5 nm-diameter pore in Figure 5d. Interestingly, this plot clearly shows that the ∆*I* induced by 50 nt ssDNA was larger than that induced by 20 nt ssDNA. In Figure 5e, Gaussian distribution was used to fit the ∆*I* histograms, and the fitting peaks for 20 nt and 50 nt ssDNA were 203.7 pA and 409.8 pA, respectively. This indicates that the 50 nt ssDNA occupied a larger volume in the pore than the 20 nt ssDNA. The short ssDNA in the large-sized pore may not be ideally parallel to the pore axis. For instance, the length of 20 nt ssDNA is only 6.8 nm, which is smaller than the pore diameter (11.5 nm). Driven by EOF, it could enter into the pore with any orientation. However, for a relatively long ssDNA, its orientation is restricted by the pore size, and the incline of the long ssDNA in the pore induces larger ∆*I*. This could be the reason why ∆*I* depends on the chain length of the ssDNA. Figure 5f shows histograms of dwell time for the electroosmotic translocation events. Events with dwell time larger than 0.5 ms were more frequent for 50 nt ssDNA than for 20 nt ssDNA. This signifies that 50 nt ssDNA is more likely to interact with the pore wall because its length is larger than the pore diameter. In addition, the distributions of measured dwell-time were fitted by a 1D drift-diffusion model [54,55,56]:

(3) $$F_{1}\left(t\right)=\frac{l}{\sqrt{4\pi Dt^{3}}}e^{-{\left(l-vt\right)}^{2}/4Dt}$$

where *D* is the diffusion coefficient and *v* refers to the velocity of the ssDNA passing through the pore. The calculated *D*_20nt_ (0.441 nm^2^/μs) was larger than the calculated *D*_50nt_ (0.382 nm^2^/μs), which agrees with the Stokes–Einstein equation [57,58]. The value of *v*_20nt_ was equal to 104.9 nm/ms, which was also larger than *v*_50nt_ (98.25 nm/ms). This can be attributed to the fact that the 20 nt ssDNA takes less negative charge than 50 nt ssDNA, which results in a smaller *F_EP_*.

## 4. Conclusions

In this study, we demonstrated that air–plasma treatment could speed up the CDB process by orders of magnitude. Only the use of the voltages in a range of 2–7 V could steadily produce nanopores with sub-10 nm diameters. Furthermore, pore size could further enlarge to >30 nm with the use of 3 V bipolar square pulses within 10 min and without causing additional pores in the SiN_x_ membrane. The results of λ-DNA detection experiments showed clear ionic pulse signals from translocation events of λ-DNA molecules, which validates the functionality of our fabricated nanopore. In addition, we used the nanopores to distinguish short ssDNA fragments with different lengths and investigate their electrokinetic behaviors. The experimental results indicated that short ssDNA fragments could enter into the pore driven by electrophoretic force or electroosmotic force, depending on the pore size and chain length of the ssDNA. Furthermore, the current blockade and dwell time of ssDNA electroosmotic translocation events increased with the chain length.
